# Effects of variable heat rise/fall on MHD Maxwell ternary nanofluid (Copper-Alumina-Titanium Dioxide/Water) flow over a moving needle

**DOI:** 10.1038/s41598-025-10057-3

**Published:** 2025-07-12

**Authors:** Amir Abbas, Laraib Kiran, Kaouther Ghachem, Tarek Salem Abdennaji, Badr M. Alshammari, Lioua Kolsi, Ilyas Khan, M. S. Khan

**Affiliations:** 1 Department of Mathematics, Faculty of Natural Sciences and Technology, Baba Guru Nanak University, Nankana Sahib, 39100 Pakistan; 2 Department of Chemistry, University of Agriculture, Faisalabad, 39000 Pakistan; 3 Department of Industrial and Systems Engineering, College of Engineering, Princess Nourah bint Abdulrahman University, P.O.Box 84428, Riyadh, 11671 Saudi Arabia; 4 Department of Civil Engineering, College of Engineering, Northern Border University, Arar, Saudi Arabia; 5 Department of Electrical Engineering, College of Engineering, University of Ha’il, Ha’il City, 81451 Saudi Arabia; 6 Department of Mechanical Engineering, College of Engineering, University of Ha’il, Ha’il City, 81451 Saudi Arabia; 7 Department of Mathematical Sciences, Saveetha School of Engineering, SIMATS, Chennai, Tamilnadu, India; 8 Department of Civil Engineering, Kardan University, Kabul, Afghanistan; 9 Hourani Center for Applied scientific Research, Al-Ahliyya Amman University, Amman, Jordan; 10 Mathematics in Applied Sciences and Engineering Research Group, Scientific Research Center, Al-Ayen University, Nasiriyah 64001, Iraq; 11 Department of Mathematics, College of Science Al-Zulfi, Majmaah University , Al-Majmaah 11952, Saudi Arabia

**Keywords:** Ternary nanofluid, Maxwell fluid, Needle, Variable heat rise/Fall, Porous media, Chemical reaction, Applied mathematics, Computational science

## Abstract

The current study explores the impact of variable heat rise/fall on the heat and mass transfer through Maxwell Ternary Nanofluid based on Copper-Alumina-Titanium Dioxide/Water. Electrically conducting non-Newtonian Maxwell fluid flowing on a moving thin needle embedded in porous media is considered. Effects of chemical reaction parameters along with the applied magnetic field in the normal direction of the flow of fluid are incorporated. The proposed mechanism in the form of differential equations is solved using the MATLAB bvp4c solver. This study can be utilized in energy systems like nuclear and chemical reactors, where managing high heat fluxes in porous environments is essential. The unique behavior of ternary nanofluids under magnetic fields improves cooling efficiency and system stability. The computed results show that the increase in the Maxwell fluid parameter causes a reduction in the velocity field and an augmentation of temperature and mass concentration. This is due to an increase in thermal relaxation time, which takes time for the adjustment of the fluid. It is concluded that an increase in the Lorentz force due to a rising magnetic field parameter results in a temperature increase and a decrease in the fluid’s velocity. The variable heat rise and fall parameter leads to an increase in the fluid’s temperature. An increase in the nanoparticle volume fraction results in elevated temperature and concentration distributions. Moreover, the Nusselt number increases with higher Prandtl numbers, while the Sherwood number decreases as the chemical reaction parameter grows. The main outcome of this current study for the case of the ternary nanofluid is that the overall thermal performance of the fluid is improved, which serves the purpose of the proposed study.

## Introduction

**Fig. 1:**
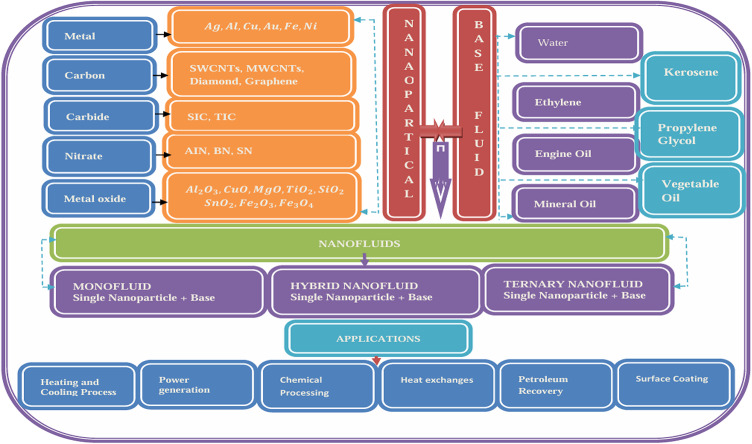
Ternary-Hybrid-Nanofluid Model.

Ternary hybrid nanofluids have significantly advanced heat transfer technology by providing exceptional improvements in thermal conductivity, viscosity, and stability. These fluids offer potential breakthroughs in energy efficiency and thermal control across sectors such as aerospace, electronics, biomedicine, and automotive engineering. Over the past few decades, researchers have devoted considerable efforts to developing optimal nanofluids that offer improved performance. Ternary hybrid nanofluids, which involve the combination of two or more distinct nanoparticles, have emerged as a promising extension of conventional single nanofluids. Extensive studies have consistently demonstrated that hybrid nanofluids exhibit superior performance characteristics compared to their single-component counterparts. The research community focused a lot on the study of such types of fluids. Hussein et al.^1^ conducted a study on the heat transfer in a ternary nanofluid comprising alumina, copper, and silica/titania nanoparticles suspended in water on a porous shrinking disk. The Lorentz forces, suction, and Joule heating effects are encountered in their study. From their study, they confirmed that alumina-copper-titania/water-based ternary nanofluid appeared as of best candidate for the thermal performance in cooling systems and industrial management applications. Ouyang et al.^2^ conducted a numerical study of tri-hybrid nanofluid flow over a moving wedge, taking into account viscous dissipation, Joule heating, and magnetic force. They computed the solution of the unsteady model. They concluded that this study proved to be an effective work for thermal management systems incorporating the time effects. Recent studies have investigated the thermal properties of various nanofluids, including mono, hybrid, and ternary hybrid nanofluids, with a focus on their application in photovoltaic/thermal systems^3^. Additionally, research has explored the effects of suction and dual-stretching on the flow behavior of ternary-hybrid nanofluids^4^, as well as the heat transfer characteristics of conventional and modified hybrid nanofluids^5^. A series of investigations have been conducted on the thermal and flow characteristics of nanofluids, including the influence of nonlinear thermal radiation on magnetohydrodynamic flow^6^ and the unsteady flow behavior of ternary hybrid nanofluids^7^. Abbas et al.^8^ presented a numerical simulation of the Darcy-Forchheimer flow of a ternary hybrid nanofluid, modeled using the Casson fluid framework, with consideration of melting heat transfer and local thermal non-equilibrium effects. Jeelani and Abbas^9^ gave a comprehensive computational analysis of the Maxwell non-Newtonian Maxwell hybrid nanofluid, which is based on ethylene glycol, incorporates the effects of a magnetic field, and suction in a porous space. Jeelani and Abbas^10^ extended the work (given in^9^), incorporating the combined effects of chemical reaction and solar radiation along with Lorentz force in Maxwell Hybrid Nanofluid past the permeable and inclined sheet in a porous space. Jeelani and Abbas^11^ extended the work (given in^10^), incorporating the combined effects of chemical reaction, heat generation, and solar radiation along with the Lorentz force in Maxwell Hybrid Nanofluid past the permeable inclined sheet in a porous space. Ashwinkumar^12^ explored the study of a hybrid nanofluid based on AA7075 + AA7072/methanol along the moving thin needle using the Newtonian model. They incorporated the non-uniform heat rise and fall, magnetic field effects, and chemical reaction. Abbas et al.^13^ focused their attention on the non-Newtonian Williamson hybrid nanofluid flow along the pass, modeling a thin needle. The impact of porosity of the media, non-uniform heat rise/fall, and magnetic force was assumed in their study. Investigations on the flow of the boundary layer past a thin needle with a constant temperature considered parallel to the moving stream were carried out by Ishak et al.^14^. The pictorial way of nanofluid exploration is given in Fig. 1.

In a boundary layer flow, these non-uniform heat sources or sinks influence how temperature gradients develop in the fluid. Heat transfer within the boundary layer is governed by the thermal conductivity of the fluid, the velocity profile (since temperature gradients are coupled with the velocity profile), and the temperature gradient created by the heat source or sink. When heat is added or removed non-uniformly, this creates local variations in the temperature gradient, which may affect the thickness of the boundary layer. If the heat source is more intense in one region, it will likely lead to a steeper temperature gradient. The smaller thermal boundary layer in that region can influence the overall heat transfer characteristics. In^15–17^, the researchers highlighted the research on the overall influence of non-uniform heat source/rise and sink/fall on the fluid flow, considering transient and non-transient effects within the porous space. Siddiqui et al.^18^ contributed to the flow of the Maxwell nanofluid along the melting surface, considering the entropy optimization and magnetic force effects. Khan et al.^19^ investigated the fractional analysis of the nanofluid flow using aluminum oxide as nanoparticles with an effective Prandtl number.

In the paragraph just above, the major tilt of the literature was on the variable heat rise and fall influence, and now in the current paragraphs, the research works indicate the physical needs of chemical reactions in the transportation of heat and fluid flow in industrial, environmental, and engineering sectors. Recent advancements in thermal and chemical transport systems demand the development of advanced fluid models capable of capturing complex interactions such as nonlinear rheology, thermal enhancement, and reactive mass transfer. Integrating chemical reaction effects into a Maxwell ternary nanofluid framework offers a more realistic and comprehensive representation of reactive transport processes in porous and magnetized media. This coupling captures the interplay between elastic fluid forces, nanoparticle-enhanced thermal transport, and reaction-induced concentration shift, a combination rarely addressed in existing studies. Therefore, the present research fills a critical gap by developing and solving a model that unites these phenomena under a unified, physically motivated framework, with potential real-world implications in energy systems, biomedical devices, and chemical engineering processes.

Many studies regarding ternary nanofluids carrying the benefits of chemical reactions have been published in^20–25^, which disclosed several potential benefits of them scientifically. By integrating the Maxwell viscoelastic model with a hybrid nanofluid formulation and incorporating chemical reaction dynamics, the present work aims to develop a more physically representative and industrially relevant model. The study addresses the critical interactions among momentum diffusion, elastic deformation, thermal enhancement via nanoparticles, and reactive solute transport, providing insights that are both theoretically novel and practically impactful. The above-highlighted published literature leaves research gaps, which are leading to the following key findings and novelty.

The literature survey reflects that many studies on the ternary nanofluids have been carried out due to potential applications in the engineering and industrial fields. This motivated the authors to conduct the current study that encircles the combined effects of the chemical reaction and magnetic force effects of the Maxwell ternary nanofluid $\:\:(Cu$-$\:A{l}_{2}{O}_{3}$-$\:Ti{O}_{2}$/Water) flow. The fluid flow is induced due to the movement at a constant speed of the thin needle embedded in a porous medium. The effects of non-uniform heat rise and fall on the fluid flow and heat transfer are considered in the current model. This study with these combined effects has not been published before this one.

## Problem formulation

Consider 2-D incompressible, steady, and viscous fluid flow past a moving thin needle. The ternary nanofluid based on $\:Cu$-$\:A{l}_{2}{O}_{3}$-$\:Ti{O}_{2}$/Water is considered in the current study. The magnetic field and chemical reaction effects are incorporated in the current formulation. The Maxwell ternary nanofluid flow in the porous media is taken. The variable thickness of the needle is taken into consideration. Cylindrical coordinate system $\:(x,r)$ has been assigned to the axial and radial directions, respectively. The surface of the thin needle is kept at a constant temperature.

**Fig. 2:**
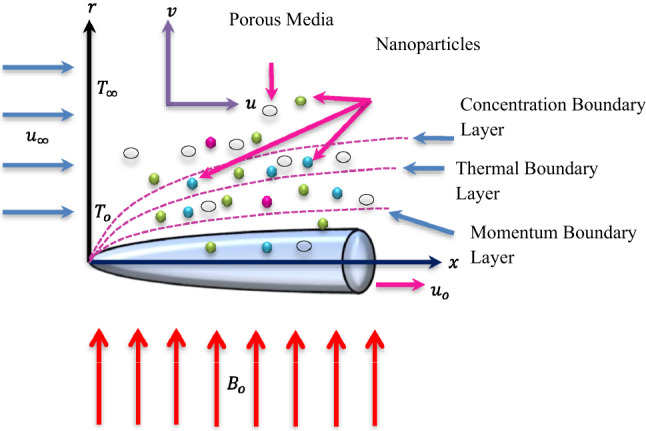
Flow structure.

The flow configuration is highlighted in Fig. 2.

The rheological governing equations are as follows:

1 $$\:T\:=\:-\:pI\:+\:S,$$

where $\:T$ stands for Cauchy stress tensor, $\:S$ defines an extra stress tensor, $\:p$ is the pressure and $\:I$ be the identity tensor.

2 $$\:S+{\lambda}_{1}\left(\frac{dS}{dt}-LS-S{L}^{T}\right){={\mu\:}_{thnf}A}_{1}.$$

Here, $\:{A}_{1}$designates the first-order Rivlin–Ericksen tensor and $\:{\lambda}_{1}$ is the relaxation time. The value of $\:{A}_{1}$ can be determined as

3 $$\:{A}_{1}=L+{L}^{T}=\left(\begin{array}{cc}2\frac{\partial\:u}{\partial\:x}&\:\frac{\partial\:u}{\partial\:r}+\frac{\partial\:v}{\partial\:x}\\\:\frac{\partial\:u}{\partial\:r}+\frac{\partial\:v}{\partial\:x}&\:2\frac{\partial\:v}{\partial\:r}\end{array}\right).$$

Multiplying the momentum equation of the Maxwell fluid by $\:\left(1+{\lambda\:}_{1}\frac{D}{Dt}\right)$ and utilizing Eq. 2, we acquire

4 $$\:{\rho\:}_{hnf}\left(1+{\lambda\:}_{1}\frac{D}{Dt}\right)={\mu\:}_{hnf}div\left({A}_{1}\right)$$

Where $$\:\frac{D}{Dt}=\frac{\partial\:}{\partial\:t}+\left(\varvec{V}\cdot\:\nabla\:\right)-L-{L}^{T}$$.

The governing equations describing the physical flow problem for Maxwell hybrid nanofluid are written as^1,13,14^:

5 $$\:\frac{\partial\:}{\partial\:x}\left(ru\right)+\frac{\partial\:}{\partial\:r}\left(rv\right)=0,$$

6 $$\begin{aligned} &\left(u\frac{\partial\:u}{\partial\:x}+v\frac{\partial\:u}{\partial\:r}\right)=\frac{{\mu\:}_{\frac{thnf}{{\mu\:}_{f}}}}{{\rho\:}_{\frac{thnf}{{\rho\:}_{f}}}}\frac{{\mu\:}_{f}}{{\rho\:}_{f}}\:\frac{1}{r}\frac{\partial\:}{\partial\:r}\left(r\frac{\partial\:u}{\partial\:r}\right)-{\lambda\:}_{1}\:\left({u}^{2}\frac{{\partial\:}^{2}u}{\partial\:{x}^{2}}+{v}^{2}\frac{{\partial\:}^{2}u}{\partial\:{r}^{2}}+2uv\frac{{\partial\:}^{2}u}{\partial\:x\partial\:r}\right)\\ & \quad-\frac{\frac{{\sigma}_{thnf}}{{\sigma\:}_{f}}}{{\rho\:}_{\frac{thnf}{{\rho\:}_{f}}}}\frac{{\sigma\:}_{f}}{{\rho\:}_{f}}{B}_{o}^{2}u-\frac{{\mu\:}_{\frac{thnf}{{\mu\:}_{f}}}}{{\rho\:}_{\frac{thnf}{{\rho\:}_{f}}}}\frac{{\mu\:}_{f}}{{\rho\:}_{f}}\frac{u}{{K}_{1}}\end{aligned}$$

7 $$\:{\left(\rho\:{C}_{p}\right)}_{thnf}\left(u\frac{\partial\:T}{\partial\:x}+v\frac{\partial\:T}{\partial\:r}\right)={k}_{thnf}\frac{1}{r}\frac{\partial\:}{\partial\:r}\left(r\frac{\partial\:T}{\partial\:r}\right)+{q}^{{\prime\prime\prime}}$$

8 $$\:\left(u\frac{\partial\:C}{\partial\:x}+v\frac{\partial\:C}{\partial\:r}\right)={D}_{B}\frac{1}{r}\frac{\partial\:}{\partial\:r}\left(r\frac{\partial\:C}{\partial\:r}\right){-K}_{1}^{*}\left(C-{C}_{\infty\:}\right).$$

The necessary conditions for Eqs. (5–8) at the boundary are described as below:

9 $$\:\left.\begin{array}{c}u={u}_{o},\:\:v=0,\:\:T={T}_{o},\:\:C={C}_{o}\:\:at\:r=R\left(x\right)\\\:u\to\:{u}_{\infty\:},\:\:T\to\:{T}_{\infty\:},\:\:C\to\:{C}_{\infty\:}\:\:as\:r\to\:\infty\:\end{array}\right\}.$$

Here, the velocity field is defined by $\:\left[\varvec{V}(u\left(x,r\right),v\left(x,r\right),0\right]$, where $\:u$ and $\:v$ respectively denote the velocity components along axial and radial axes. $\:T\left(x,r\right)$ and $\:C\left(x,r\right)$ respectively define the temperature of Maxwell ternary nanofluid and nanoparticle concentration, and $\:{K}_{1}$ is the porosity of a porous media. The notation, $\:{D}_{B}$ is used as an abbreviation for Brownian diffusion. The radius of the paraboloid needle is related to $\:R\left(x\right)={\left(\frac{{\nu\:}_{f}xc}{{U}_{o}}\right)}^{1/2}$. $\:\rho\:$, $\:\mu\:$, $\:\sigma\:$, $\:{C}_{p}$ and $\:k$ highlight the physical properties involving density, viscosity coefficient, electrical conductivity, specific heat coefficient, and thermal conductivity for the ternary nanofluid as indicated by their subscript $\:hnf$. Expression of $\:{q}^{{\prime\prime\prime}}$ represents the non-uniform heat variation (rise/fall) and is mathematically understood as$\:\:\frac{{k}_{f}{U}_{o}}{{\nu\:}_{f}x}\left\{A\left({T}_{o}-{T}_{\infty\:}\right){f}^{{\prime\:}}+B\left(T-{T}_{\infty\:}\right)\right\}$. Where $\:{K}_{1}^{*}={\stackrel{\sim}{K}}_{1}^{*}/\stackrel{\sim}{x}$ is abbreviated as dimensional chemical reaction rate, where $\:{K}_{1}^{*}$ is the coefficient of a chemical reaction.

## Solution methodology

This section is intended to explain in detail the method applied to the system of Eqs. (5–8) by integrating the boundary conditions defined by Eq. (9). The approach adopted transforms the differential equations from their partial form into ordinary form before proceeding to the numerical solution. The solutions by similarity are first carried out, then the system of equations is processed by the bvp4c numerical solver in the subsequent parts.

### Similarity analysis

For the resolution of the aforementioned governing Eqs. (5–8), along with their respective boundary conditions, a non-dimensionalization approach is employed. This involves the utilization of similarity transformations and the stream function to get the dimensionless equations. In alignment with the procedures delineated in^14^, the corresponding similarity variables are:

10 $$\:\psi\:={\nu\:}_{f}xf\left(\eta\:\right),\:\:\theta\:\left(\eta\:\right)=\frac{T-{T}_{\infty\:}}{{T}_{o}-{T}_{\infty\:}},\:\:\phi\:\left(\eta\:\right)=\frac{C-{C}_{\infty\:}}{{C}_{o}-{C}_{\infty\:}},\:\:\eta\:=\frac{{U}_{o}{r}^{2}}{{\nu\:}_{f}x},$$

In Eq. (10), $\:{U}_{o}={u}_{o}+{u}_{\infty\:}$, $\:{\nu\:}_{f}$ and $\:\eta\:$ indicates composite velocity, kinematic viscosity coefficient, and transformed similarity variable. The flow under consideration is streamlined; therefore, the decomposed velocity components $\:(u,v)$ can be written as

11 $$\:u={r}^{-1}{\psi\:}_{r},v=-{r}^{-1}{\psi\:}_{x}.$$

The correlations indicating thermo-physical characteristics of ternary nanofluids are as follows:12

The corresponding volume fractions of nanoparticles $\:Cu$-$\:A{l}_{2}{O}_{3}$-$\:Ti{O}_{2}$ are respectively signified by $\:{\phi}_{1}$, $\:{\phi}_{2}$, and $\:{\phi}_{3}$. Utilizing Eqs. (10 and 11), the dimensionless ordinary differential equations are expressed as

13 $$\begin{aligned} &\frac{\frac{2{\mu\:}_{thnf}}{{\mu\:}_{f}}}{\frac{{\rho\:}_{thnf}}{{\rho\:}_{f}}}\left(\eta\:{f}^{{\prime\prime\prime}}+{f}^{{\prime\prime}}\right)+f{f}^{{\prime\prime}}-\beta\:\left[\left({f}^{2}{f}^{{\prime\prime}}+{\eta\:}^{2}{{f}^{{\prime\:}}}^{2}{f}^{{\prime\prime}}\right)+2\eta\:\left({f}^{2}{f}^{{\prime\prime\prime}}+f{f}^{{\prime}}{f}^{{\prime\prime}}\right)\right]\\& \quad -\frac{\frac{{\sigma\:}_{thnf}}{{\sigma\:}_{f}}}{{\rho\:}_{\frac{thnf}{{\rho\:}_{f}}}}\frac{1}{2}\:M{f}^{{\prime}}-\frac{1}{2}\frac{{\mu\:}_{\frac{thnf}{{\mu\:}_{f}}}}{{\rho\:}_{\frac{thnf}{{\rho\:}_{f}}}}Da{f}^{{\prime}}=0, \end{aligned}$$

14 $$\:{\theta}^{{\prime\prime}}+\left(\frac{1}{\eta\:}+\frac{{k}_{f}Pr}{{k}_{thnf}2\eta\:}\frac{{\left(\rho\:{C}_{p}\right)}_{thnf}}{{\left(\rho\:{C}_{p}\right)}_{f}}f\right){\theta}^{{\prime\:}}+\frac{{k}_{f}}{{k}_{thnf}4\eta\:}\left(A{f}^{{\prime\:}}+B\theta\:\right)=0,$$

15 $$\:{\phi}^{{\prime\prime}}\eta\:+\left(1+\frac{Sc}{2}f\right){\phi\:}^{{\prime}}-\frac{1}{4}ScKr\phi=0,$$

With boundary conditions in dimensionless form are:

16 $$\:\left.\begin{array}{c}f\left(c\right)=\frac{\delta\:c}{2},\:\:{f}^{{\prime}}\left(c\right)=\frac{\delta\:}{2},\:\:\theta\:\left(c\right)=1,\:\:\phi\:\left(c\right)=1\:\text{a}\text{t}\:\eta\:=c\\\:{f}^{{\prime}}\left(\eta\:\right)\to\:\frac{1-\delta\:}{2},\:\:\theta\:\left(\eta\:\right)\to\:0,\:\:\phi\left(\eta\:\right)\to\:0\:as\:\eta\:\to\:\infty\:\end{array}\right\}$$

As a result of transformations, the continuity equation can be identically satisfied. Equations (13–15) along with their boundary given in (16) is the eventually obtained dimensionless boundary value problem in which the physical parameters of interest in dimensionless form can be stated as the magnetic parameter $\:\left\{M=\frac{{\sigma\:}_{f}{B}_{0}^{2}x}{{U}_{o}{\rho\:}_{f}}\right\}$, Maxwell parameter $\:\left\{\beta\:=\frac{{\lambda\:}_{1}{\nu\:}_{f}}{{r}^{2}}\right\}$, porosity parameter $\:\left\{Da=\frac{{\nu\:}_{f}x}{{K}_{1}{U}_{o}}\right\}$ velocity ratio $\:\left\{\delta\:=\frac{{u}_{o}}{{U}_{o}}\right\}$, Prandtl number $\:\left\{Pr=\frac{{\nu\:}_{f}}{{\alpha\:}_{f}}\right\}$, Schmidt number $\:\left\{Sc=\frac{{\nu\:}_{f}}{{D}_{B}}\right\}$, and $\:A$ and $\:B$ be irregular heat rise and fall parameters, and $\:Kr=\frac{{K}_{1}^{*}}{{U}_{o}}$ a chemical reaction parameter, respectively, and the Chemical reaction parameter $\:Kr$. The dimensionless system of equations is more efficacious in fluid problems as it generalizes any flow problem, and it can be used to describe many-dimensional problems rather than any particular one. Table 1 represents the thermophysical properties of nanoparticles and the base fluid.

Table 1 lists thermophysical characteristics of the base fluid and the nanoparticles as used by^1^.

**Table 1:**
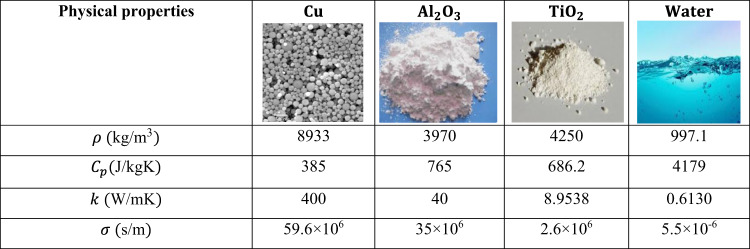
Properties of nanoparticles and water.

### Principal quantities of interest

Furthermore, after following, the dimensional skin friction coefficient $\:R{e}^{1/2}{C}_{f}$, local Nusselt number $\:R{e}^{-1/2}N{u}_{x}$ and local Sherwood number $\:R{e}^{-1/2}S{h}_{x}$ being the practical quantities of interest are written below,

The assessment of key physical quantities of interest has been carried out, including the local Nusselt number $\:N{u}_{x}$ which characterizes the heat transfer rate, the skin friction coefficient $\:{C}_{f}$ representing the shear stress between the fluid and the surface; and the local Sherwood number Sh_​, indicating the mass transfer rate. These dimensionless parameters play a critical role in understanding the transport phenomena involved and are therefore discussed in detail in the following sections.

17 $$\begin{aligned} &{C}_{f}=\frac{1}{{\rho\:}_{f}{U}_{0}^{2}}{\left(\frac{{\mu\:}_{hnf}}{{\mu\:}_{f}}\frac{\partial\:u}{\partial\:r}-{\lambda\:}_{1}(2uv\frac{{\partial\:}^{2}u}{\partial\:x\partial\:x}+{v}^{2}\frac{{\partial\:}^{2}u}{\partial\:{r}^{2}})\right)}_{r=a}, \\ &\:N{u}_{x}=-\frac{{xk}_{hnf}}{{k}_{f}\left({T}_{o}-{T}_{\infty\:}\right)}{\left(\frac{\partial\:T}{\partial\:r}\right)}_{r=a}, \\ & \:S{h}_{x}=-\frac{x}{\left({C}_{o}-{C}_{\infty\:}\right)}{\left(\frac{\partial\:C}{\partial\:r}\right)}_{r=a},\end{aligned}$$

To make (17) dimensionless, we utilize (10–11), and then we have:

18 $$\:\left.\begin{array}{c}\frac{{Re}_{x}^{1/2}{C}_{f}}{4{c}^{1/2}}=\left({\frac{{\mu\:}_{hnf}}{{\mu\:}_{f}}{f}^{{\prime\prime}}\left(c\right)-\beta\:f}^{2}\left(a\right){f}^{{\prime}{\prime}}\left(c\right)-2ff ^{\prime}\left(c\right)\right)\\\:\frac{{Re}_{x}^{-1/2}N{u}_{x}}{2{c}^{1/2}}=-\frac{{k}_{hnf}}{{k}_{f}}{\theta\:}^{{\prime\:}}\left(c\right)\\\:\frac{{Re}_{x}^{-1/2}S{h}_{x}}{2{c}^{1/2}}=-{\phi}^{{\prime}}\left(c\right)\:\end{array}\right\},$$

The value ${Re}_{x}=\frac{{U}_{o}x}{{\nu\:}_{f}}$ in the above equation is named local Reynolds number.

### Solution technique

Based on the collocation method, the bvp4c solver is employed for the solution of Eqs. (13–15) and for Eq. (18) with their boundary conditions provided in Eq. (16). These equations along with their boundary conditions are directed to input in MATLAB bvp4c solver to extract the numerical solution in form of velocity, thermal and mass distribution, along with skin friction coefficient, local Nusselt number and local Sherwood number. The numerical computations are carried out using MATLAB built-in boundary value problem solver bvp4c. In the simulations, the computational domain is truncated at $\:\eta\:=\infty\:$, to ensure solution convergence, and the axes are scaled appropriately for clear figure visibility. The bvp4c solver is based on the finite difference method and implements a three-stage Lobatto III collocation formula. This method yields a $\:{C}^{1}$, continuous solution with uniform fourth-order accuracy over the integration domain. The collocation technique employed by bvp4c divides the domain into subintervals using a mesh of points. A global system of nonlinear algebraic equations is formed by enforcing the boundary conditions and the collocation conditions across these subintervals. The solver then estimates the error in each subinterval and, if the solution does not meet the specified tolerance, it refines the mesh adaptively and iteratively resolves the system. The method requires an initial mesh and an initial guess for the solution values at the mesh points. The numerical results obtained using this method are compared with previously published data, demonstrating excellent agreement. This comparison confirms both the accuracy and the validity of the present results.

These equations are further progressed as follows

19 $$\:\text{Y}\left(1\right)=f,\:\text{Y}\left(2\right)={f}^{{\prime}},\:\text{Y}\left(3\right)={f}^{{\prime}{\prime}},\:\text{Y}\left(4\right)=\theta,\:\text{Y}\left(5\right)={\theta}^{{\prime}},\text{Y}\left(6\right)=\phi,\:\text{Y}\left(7\right)=\phi^{\prime},$$

20 $$\begin{aligned} \:\text{Y}\text{Y}1=-\frac{ \begin{array}{l} \left[\left\{\left(2*\frac{{\mu\:}{\frac{thnf}{{\mu\:}{f}}}}{{\rho\:}{\frac{thnf}{{\rho\:}{f}}}}-\text{Y}\left(3\right)\right)-\beta\:\left(\text{Y}{\left(1\right)}^{2}+{\eta\:}^{2} * {\text{Y}\left(2\right)}^{2}+2*\eta\: * \text{Y}\left(2\right)\right)\right\} \right. \\ \qquad \left. * \text{Y}\left(3\right) -\frac{\frac{{\sigma\:}{thnf}}{{\sigma\:}{f}}}{{\rho\:}{\frac{thnf}{{\rho\:}{f}}}} * \frac{1}{2}* M*\text{Y}\left(2\right)-\frac{1}{2} * \frac{{\mu\:}{\frac{thnf}{{\mu\:}{f}}}}{{\rho\:}{\frac{thnf}{{\rho\:}{f}}}}Da * \text{Y}\left(2\right)\right] \end{array} }{\left(2\frac{{\mu\:}{\frac{thnf}{{\mu\:}{f}}}}{{\rho\:}{\frac{thnf}{{\rho\:}{f}}}}-2*\beta\: * \text{Y}{\left(1\right)}^{2} * \eta\:\right)} \end{aligned}$$

21 $$\:\text{Y}\text{Y}2=-\left(\frac{1}{\eta\:}+\frac{{k}_{f}*Pr*}{{k}_{thnf}*2*\eta\:}\frac{{\left(\rho\:{C}_{p}\right)}_{hnf}}{{\left(\rho\:{C}_{p}\right)}_{f}}*\text{Y}\left(1\right)\right)*\text{Y}\left(5\right)-\frac{{k}_{f}}{{k}_{thnf}*4*\eta\:}\left({A}^{*}\text{Y}\left(2\right)+{B}^{*}\text{Y}\left(4\right)\right)$$

22 $$\:\text{Y}\text{Y}3=-\frac{\left(1+\frac{Sc}{2}\text{*}\text{Y}\left(1\right)\right)\text{*}\text{Y}\left(6\right)-\frac{1}{4}\text{*}Sc\text{*}Kr\text{*}Y\left(6\right)}{\eta},$$

Boundary conditions

23 $$\:\left.\begin{array}{c}\text{Y}\left(1\right)=\frac{\delta\:c}{2},\:\text{Y}\left(2\right)=\frac{\delta\:}{2},\:\text{Y}\left(4\right)=1,\text{Y}\left(6\right)=1\:\:\text{a}\text{t}\:\eta\:=c\\\:\text{Y}\left(2\right)\to\:\frac{1-\delta\:}{2},\:\text{Y}\left(4\right)\to\:0,\text{Y}\left(6\right)\to\:0\:\:\:\:\text{a}\text{s}\:\eta\:\to\:\infty\:\end{array}\right\}.$$

The system of non-linear first-order differential equations is implemented in MATLAB using the bvp4c solver to obtain the final numerical solutions. The resulting tabular data and graphical outputs are appropriately incorporated into the manuscript. At each spatial location, the iterative process proceeds until the convergence criterion of $\:{10}^{-5}$ met for all variables, ensuring that the boundary conditions are satisfied both at the surface and in the asymptotic far-field. The convergence criteria for $\:{f}^{{\prime}},\theta,\:\phi$ at each iteration step, based on a 20 × 20 mesh size, are defined as follows:

24 $$\:\text{max}\left|{f}^{{\prime\:}}\right|+\text{max}\left|f\right|+\text{max}\left|\theta\:\right|+\text{m}\text{a}\text{x}\left|\phi\right|\le\:{10}^{-5}.$$

The computation begins at $\:\eta=0$, and progresses downstream using an implicit scheme. As per the convergence criteria defined in Eq. (24) and the boundary conditions in Eq. (23), the numerical results shown in the graphs closely satisfy the surface constraints. Moreover, the solutions exhibit proper asymptotic behavior away from the surface, indicating that the boundary conditions are accurately fulfilled within the chosen mesh and computational domain. Notably, the CPU time for completing one iteration loop (based on two distinct values of a parameter) was approximately 32 s, highlighting the efficiency of the numerical method.

**Fig. 3:**
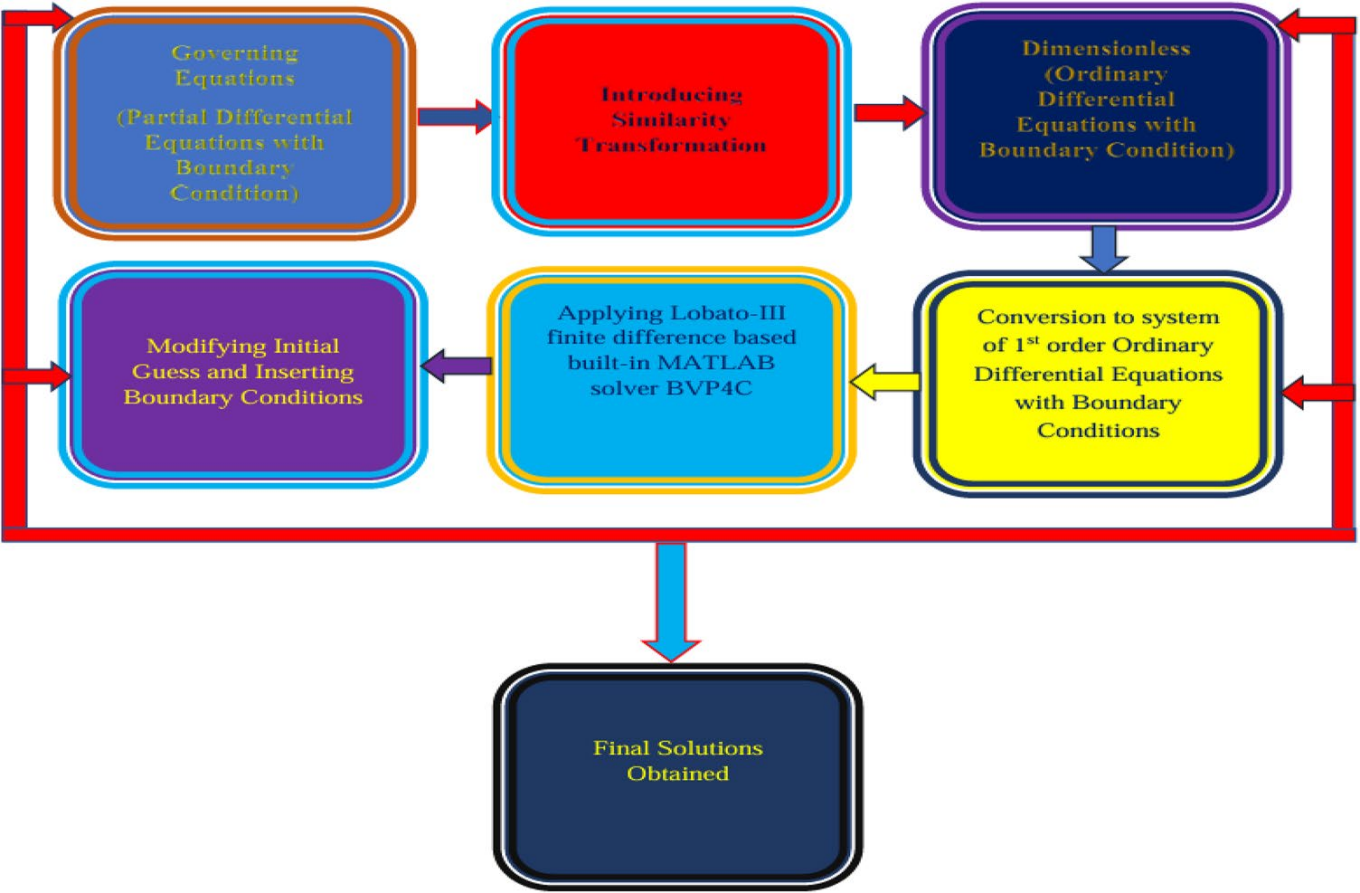
BVP4C solution technique schematic diagram.

## Results and discussion

The graphical outcomes of the problem have been generated to evaluate the velocity profile $\:f{^\prime}\left(\eta\right)$, temperature field $\:\theta\left(\eta\:\right)$, mass concentration profile $\:\phi\left(\eta\right)$, skin friction coefficient $\:{Re}_{x}^{1/2}{C}_{f}$, Nusselt number $\:{Re}_{x}^{-1/2}N{u}_{x}$, and Sherwood number $\:{Re}_{x}^{-1/2}S{h}_{x}$. The controlling parameters for which these results are calculated are the Maxwell fluid parameter $\:\beta\:$, Hartmann number $\:M$, porosity parameter $\:Da$, velocity ratio parameter $\:\delta\:$, thickness of the thin needle $\:c$, Prandtl number $\:Pr$, Schmidt number $\:Sc$, non-uniform heat rise and fall parameters $\:A$, $\:B$, chemical reaction parameter $\:Kr$, and volume fractions $\:{\phi}_{1},\:{\phi}_{2}$, and $\:{\phi}_{3}$ for $\:Cu-A{l}_{2}{O}_{3}-Ti{O}_{2}$, respectively.

### Effect of Maxwell fluid parameter on velocity, temperature, and concentration fields

**Fig. 4:**
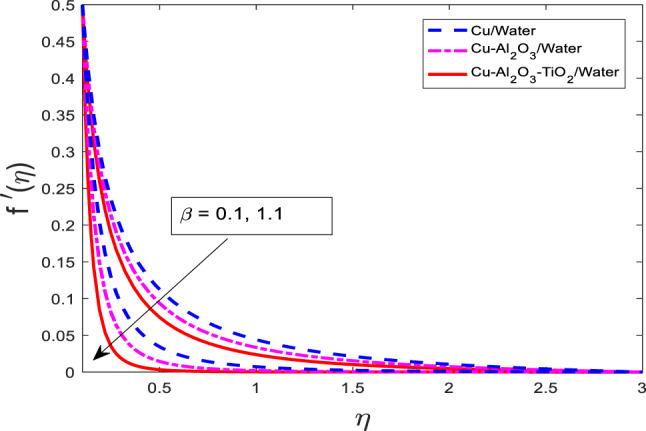
Graphical variations in $$\:f{^\prime}\left(\eta\right)$$ for $$\:Pr=6.83;\:M=1.5;\:A=0.1;\:B=3.1;\delta\:=1.0;c=0.9;$$$$\:Da\:=\:1.1;Sc\:=\:5.1;Kr\:=0.1,\:{\phi}_{1}={\phi}_{2}={\phi}_{3}=0.05$$.

**Fig. 5:**
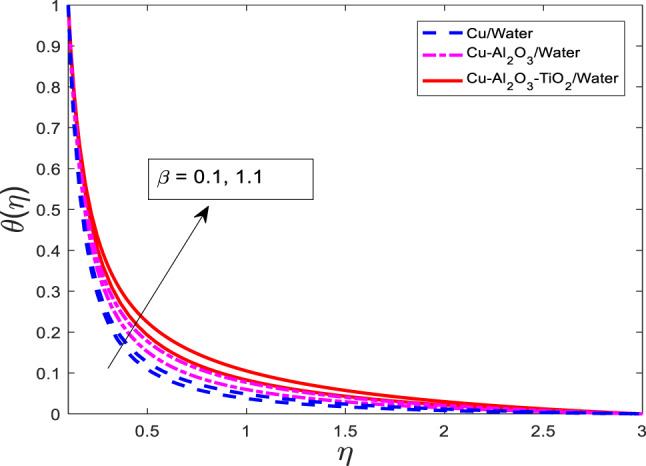
Graphical variations in $$\:f{^\prime}\left(\eta\right)$$ for $$\:Pr=6.83;\:M=1.5;\:A=0.1;\:B=3.1;\delta\:=1.0;c=0.9;\:$$$$Da=1.1;Sc\:=\:5.1;Kr\:=0.1;\:\:{\phi}_{1}={\phi}_{2}={\phi}_{3}=0.05$$.

**Fig. 6:**
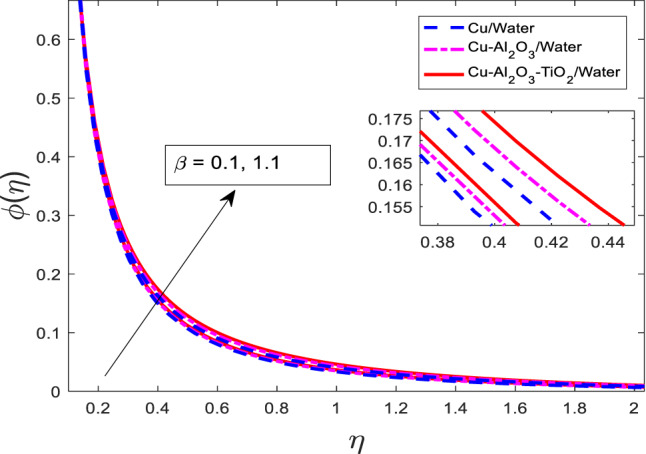
Graphical variations in $$\:f{^\prime}\left(\eta\right)$$ for $$\:Pr=6.83;\:M=1.5;\:A=0.1;\:B=3.1;\delta\:=1.0;c=0.9;\:$$$$Da=1.1;Sc\:=\:5.1;\:Kr\:=0.1;\:{\phi}_{1}={\phi}_{2}={\phi}_{3}=0.05$$.

The graphical results for $\:f{^\prime\:}$, $\:\theta\:$, and $\:\phi\:$ for increasing values of $\:\beta\:$ against three cases of nanofluid $\:Cu/$Water, hybrid nanofluid $\:Cu-A{l}_{2}{O}_{3}/$Water, and ternary nanofluid $\:Cu-A{l}_{2}{O}_{3}-Ti{O}_{2}/$Water are shown in Figs. 4, 5 and 6, respectively. When $\:\beta\:$ is varied from $\:0.1$ to $\:1.1$, physical variable $\:f{^\prime\:}$ goes down, but $\:\theta\:$, and $\:\phi\:$ go up for all three cases as portrayed in Figs. 4, 5 and 6, respectively. The graphical curves for the nanofluid $\:Cu/$Water are at a higher level than of hybrid nanofluid $\:Cu-A{l}_{2}{O}_{3}/$Water, and ternary nanofluid $\:Cu-A{l}_{2}{O}_{3}-Ti{O}_{2}/$Water in $\:f{^\prime\:}$ and opposite variations are noted in the $\:\theta\:$, and $\phi$, as reflected in Figs. 4, 5 and 6, respectively. The viscoelastic nature of the Maxwell fluid introduces additional resistance to flow, which results in the observed reduction in $\:f{^\prime\:}$.

### Effect of magnetic field parameter on velocity, temperature and concentration field

**Fig. 7:**
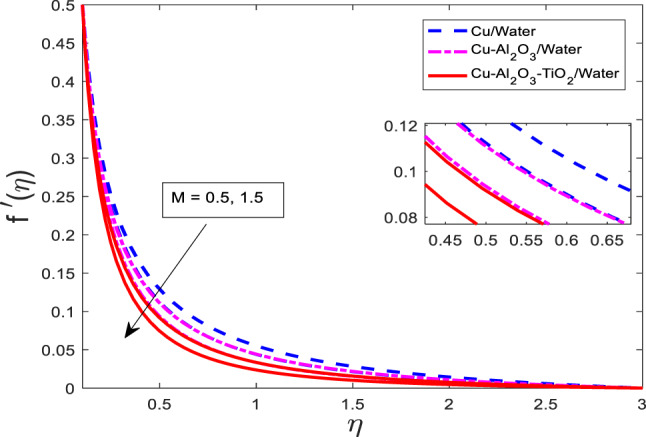
Graphical variations in $$\:f{^\prime}\left(\eta\right)$$ for $$\:Pr\:=\:6.83;\:\beta\:\:=\:0.1;\:A=0.1;\:B=3.1;\:\delta\:\:=\:1.0;\:c\:=\:0.9;\:$$$$Da\:=\:1.1;Sc\:=\:5.1;Kr\:=0.1;{\phi}_{1}={\phi}_{2}={\phi}_{3}=0.05$$.

**Fig. 8:**
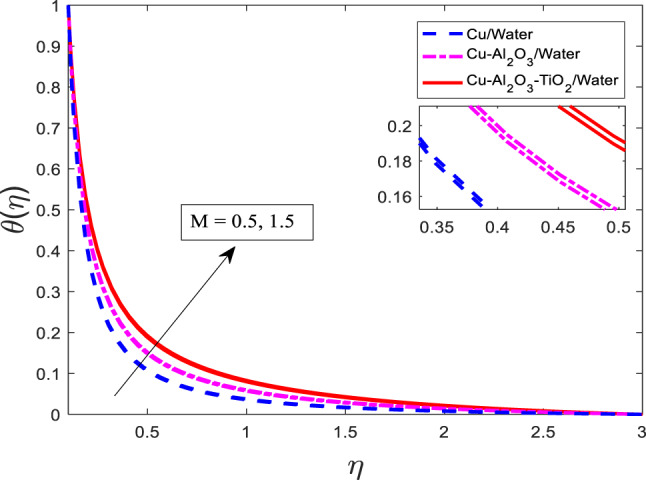
Graphical variations in $$\:\theta\left(\eta\right)$$ for $$\:Pr\:=\:6.83;\:\beta\:\:=\:0.1;\:A=0.1;\:B=3.1;\:\delta\:\:=\:1.0;\:c\:=\:0.9;\:$$$$Da\:=\:1.1;Sc\:=\:5.1;Kr\:=0.1;{\phi}_{1}={\phi}_{2}={\phi}_{3}=0.05$$.

**Fig. 9:**
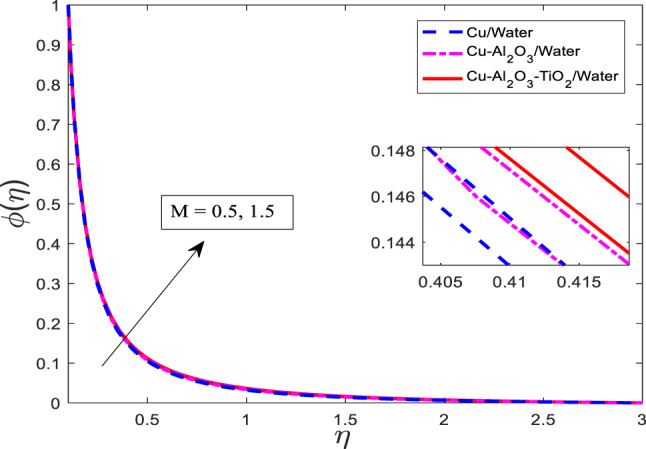
Graphical variations in $$\:\phi\left(\eta\right)$$ for $$\:Pr\:=\:6.83;\beta\:\:=\:0.1;\:A=0.1;\:B=3.1;\:\delta\:\:=\:1.0;\:c\:=\:0.9;\:$$$$Da\:=\:1.1;Sc\:=\:5.1;Kr\:=0.1;{\phi}_{1}={\phi}_{2}={\phi}_{3}=0.05$$.

Figures 7, 8 and 9 are plotted for increasing values of $\:M$ for three cases, such as nanofluid $\:Cu/$Water, hybrid nanofluid $\:Cu-A{l}_{2}{O}_{3}/$Water, and ternary nanofluid $\:Cu-A{l}_{2}{O}_{3}-Ti{O}_{2}/$Water when other parametric conditions are kept fixed. Graphical curves reflect that when $\:M$ increases, the velocity of the fluid decreases, and temperature along with concentration profile increases as shown in Figs. 7, 8 and 9, respectively. The most important physical point is that the curves for the nanofluid $\:Cu/$Water are upper boundary layer region, curves for the hybrid nanofluid $\:Cu-A{l}_{2}{O}_{3}/$Water are lower than the nanofluid $\:Cu/$Water, and curves for ternary nanofluid $\:Cu-A{l}_{2}{O}_{3}-Ti{O}_{2}/$Water are in lower region than both of the cases. This is physically correct when a single type of nanoparticle is mixed in Water, then the velocity is higher, but as the nanoparticle types are enhanced, the velocity gets weaker because the fluid becomes more viscous. Similarly, the temperature rises and the concentration gets stronger as well. The thermal performance increases with increasing kinds of mixture of nanoparticles.

### Effect of heat rise parameter on velocity, temperature, and concentration field

**Fig. 10:**
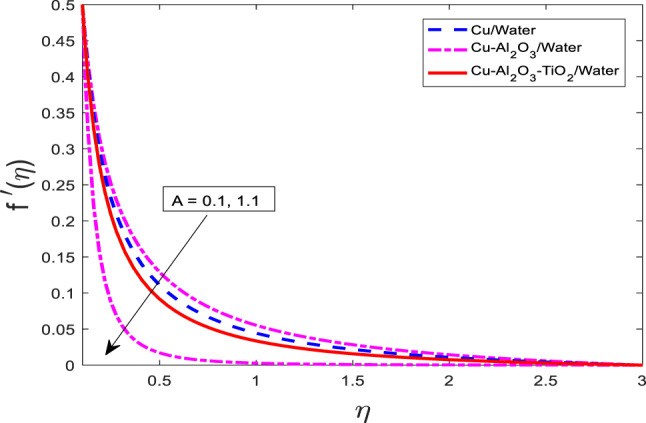
Graphical variations in $$\:f{^\prime}\left(\eta\right)$$ for $$\:Pr=6.83;\:M=0.5;\:\beta\:=0.1;\:B=3.1;\:\delta\:\:=1.0;c=0.9;\:$$$$Da=1.1;Sc\:=\:5.1;\:Kr\:=0.1;{\phi}_{1}={\phi}_{2}={\phi}_{3}=0.05$$.

**Fig. 11:**
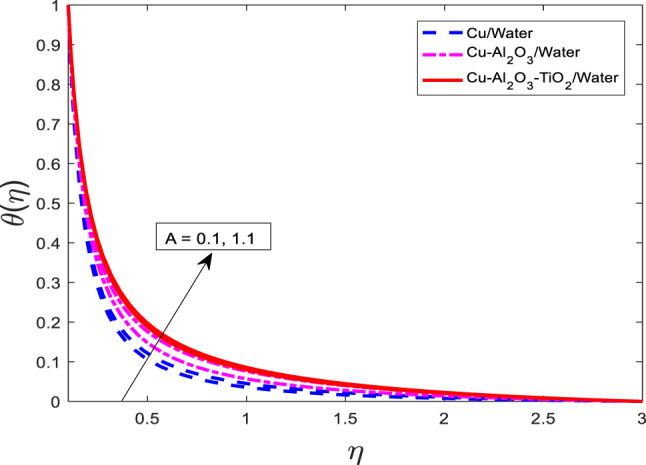
Graphical variations in $$\:\theta\left(\eta\right)$$ for $$\:Pr=6.83;\:M=0.5;\:\beta\:=0.1;\:B=3.1;\:\delta\:\:=1.0;c=0.9;\:$$$$Da=1.1;Sc\:=\:5.1;\:Kr\:=0.1;{\phi}_{1}={\phi}_{2}={\phi}_{3}=0.05$$.

**Fig. 12:**
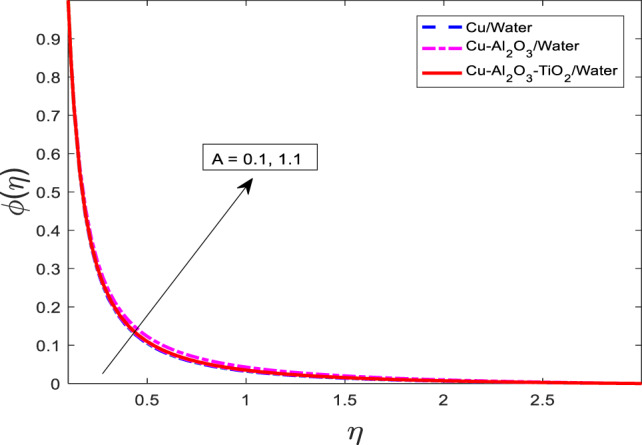
Graphical variations in $$\:\phi\left(\eta\right)$$ for $$\:Pr=6.83;\:M=0.5;\:\beta\:=0.1;\:B=3.1;\:\delta\:\:=1.0;c=0.9;\:$$$$Da=1.1;Sc\:=\:5.1;\:Kr\:=0.1;{\phi}_{1}={\phi}_{2}={\phi}_{3}=0.05$$.

Figures 10, 11 and 12 represent the graphical results for$\:f{^\prime}$, $\theta$, and $\phi$ versus increasing values of the heat rise parameter *A*. Increasing values of *A* are leading to decreasing behavior of $f{^\prime}$, and an increasing trend of $\theta$, and $\phi$ for all three kinds of fluids. Curves of $\theta$ and $\phi$ for the case of nanofluid Water are at the lowest level, and for the case of ternary nanofluid $\:Cu-A{l}_{2}{O}_{3}-Ti{O}_{2}/$Water are the highest magnitude. Physically, an increase in the heat generation parameter implies a stronger internal heat source within the fluid. This additional heat input enhances the thermal energy in the system, resulting in elevated fluid temperature and solute concentration, hence the rise in $\theta$, $\phi$. However, the increase in temperature reduces the fluid’s momentum due to intensified viscous effects and thermal diffusion, leading to a reduction in the velocity $\:f{^\prime}$.

### Effect of heat fall parameter on velocity, temperature, and concentration field

**Fig. 13:**
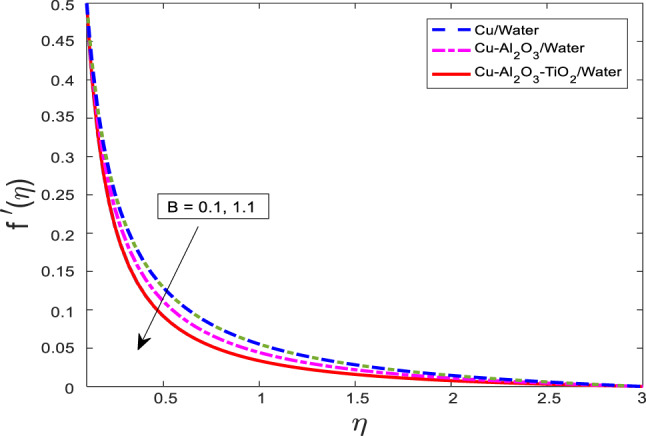
Graphical variations in $\:f{^\prime}\left(\eta\right)$ for $\:Pr=6.83;\:M=0.5;\:\beta\:=0.1;\:B=3.1;\:$ $\delta=1.0;\:c=0.9;\:Da=1.1;Sc=5.1;\:$ ${Kr=0.1;\:\phi}_{1}={\phi}_{2}={\phi}_{3}=0.05$.

**Fig. 14:**
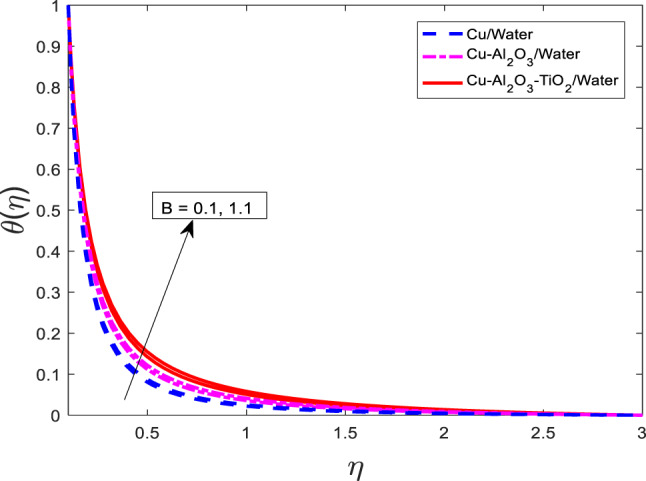
Graphical variations in $$\:\theta \left(\eta\right)$$ for $$\:Pr=6.83;\:M=0.5;\:\beta\:=0.1;\:B=3.1;\:\delta=1.0;c=0.9;\:$$$$Da=1.1;Sc=5.1;\:{Kr=0.1;\:\phi}_{1}={\phi}_{2}={\phi}_{3}=0.05$$.

**Fig. 15:**
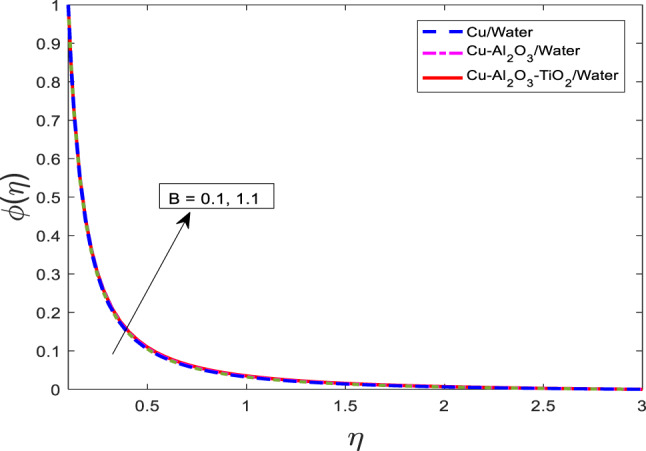
Graphical variations in $$\:\phi\left(\eta\:\right)$$ for $$\:Pr=6.83;\:M=0.5;\:\beta=0.1;\:B=3.1;\:\delta=1.0;c=0.9;\:$$$$Da=1.1;Sc\:=\:5.1;Kr\:=0.1;\:{\phi}_{1}={\phi}_{2}={\phi}_{3}=0.05$$.

Figures 13, 14, 15 depicting the non-uniform heat fall parameter $\:B$ on for $\:f{^\prime\:}$, $\:\theta\:$, and $\:\phi\:$, respectively. It has been noted that as $\:B$ increases in velocity get weaker, and temperature along concentration profiles grow rapidly for the nanofluid $\:Cu/$Water are at a higher level than hybrid nanofluid $\:Cu-A{l}_{2}{O}_{3}/$Water, and ternary nanofluid $\:Cu-A{l}_{2}{O}_{3}-Ti{O}_{2}/$Water. Physically, the parameter $\:B$ represents a spatially varying heat removal effect. As $\:B$ increases, the rate of heat extraction becomes more intense in localized regions, which paradoxically can result in enhanced thermal gradients and diffusion effects. This leads to higher thermal and solute boundary layer thicknesses, causing an increase in $\:\theta\:$ and $\:\phi\:$. Meanwhile, the increase in thermal resistance and viscous dissipation suppresses the momentum transfer, resulting in a reduction in the velocity $\:f{^\prime\:}$.

### Effect of velocity ratio parameter on velocity, temperature, and concentration field

**Fig. 16:**
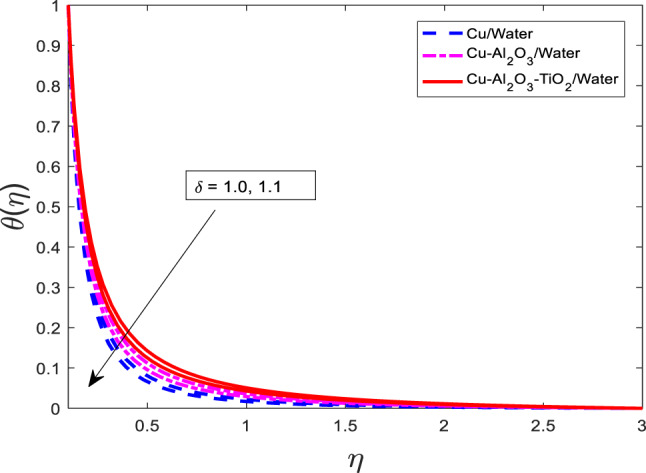
Graphical variations in $$\theta$$ for $$\:Pr=6.83;\:M=0.5;\beta\:=0.1;\:A=0.1;\:B=0.1; c=0.9;$$$$Da=1.1;Sc=5.1;Kr\:=0.1;\:{\phi}_{1}={\phi}_{2}={\phi}_{3}=0.05$$.

**Fig. 17:**
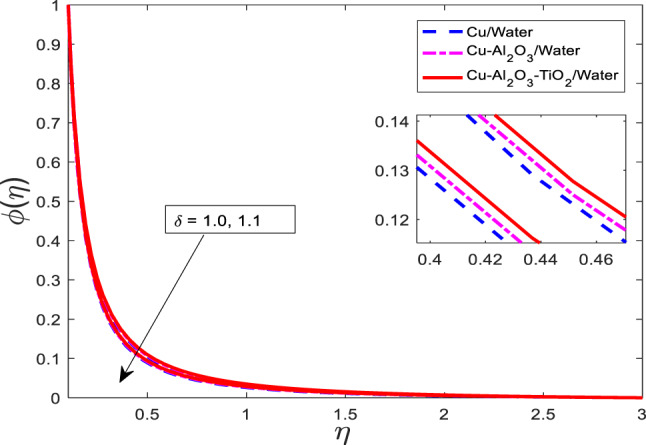
Graphical variations in $$\phi$$ for $$Pr=6.83;\:M=0.5;\beta\:=0.1;\:A=0.1;\:B=0.1; \:c=0.9;\:$$$$Da\:=1.1;Sc\:=\:5.1;Kr\:=0.1;\:{\phi}_{1}={\phi}_{2}={\phi}_{3}=0.05$$.

**Fig. 18:**
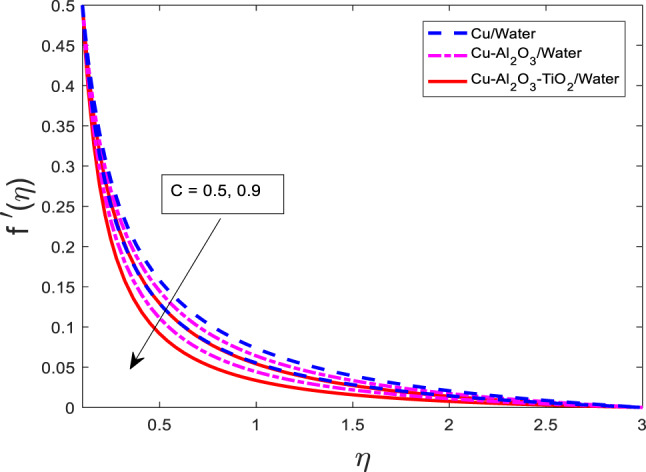
Graphical variations in $$\:f{^\prime\:}$$ $$\:Pr=6.83;\:M=0.5;\beta\:=0.1;\:A=0.1;\:B=0.1;\delta\:=1.0;\:$$$$Da=1.1;Sc\:=\:5.1;\:Kr\:=0.1;{\phi}_{1}={\phi}_{2}={\phi}_{3}=0.05$$.

Figures 16, 17, 18 highlighting the impact of the velocity ratio parameter $\:\delta\:$. When $\:\delta\:$ is enhanced, then temperature and concentration distribution are raised as displayed in Figs. 16, 17, 18, respectively. The curves of $\:\theta\:$ and $\phi$ for nanofluid $\:Cu/$Water are the lowest magnitude and for ternary nanofluid $\:Cu-A{l}_{2}{O}_{3}-Ti{O}_{2}/$Water are at the peak point. Physically, the velocity ratio parameter $\:\delta\:$ characterizes the relative strength of the stretching or shrinking surface compared to the free stream velocity. A higher value of $\:\delta\:$ implies stronger surface motion, which intensifies the boundary layer interaction and enhances thermal and mass diffusion in the fluid. As a result, both the temperature and concentration fields expand, leading to higher values of $\:\theta\:$ and $\phi$.

### Effect of the thickness of the needle on velocity, temperature, and concentration field

**Fig. 19:**
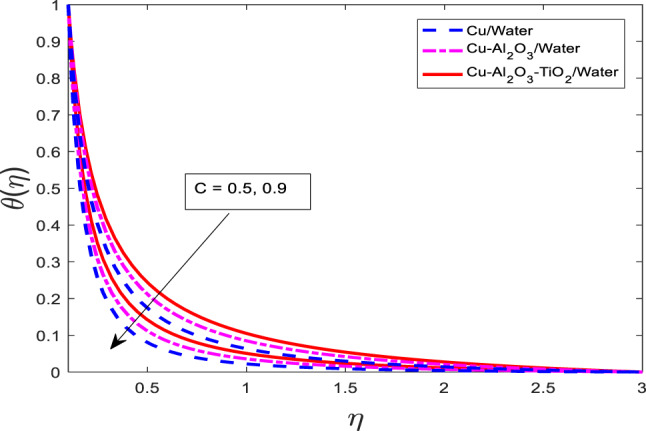
Graphical variations in $$\:\theta\:$$ $$\:Pr=6.83;\:M=0.5;\:\beta\:=0.1;\:A=0.1;\:B=0.1;\delta\:=1.0;\:$$$$Da=1.1;Sc\:=\:5.1;Kr\:=0.1;\:{\phi}_{1}={\phi}_{2}={\phi}_{3}=0.05$$.

**Fig. 20:**
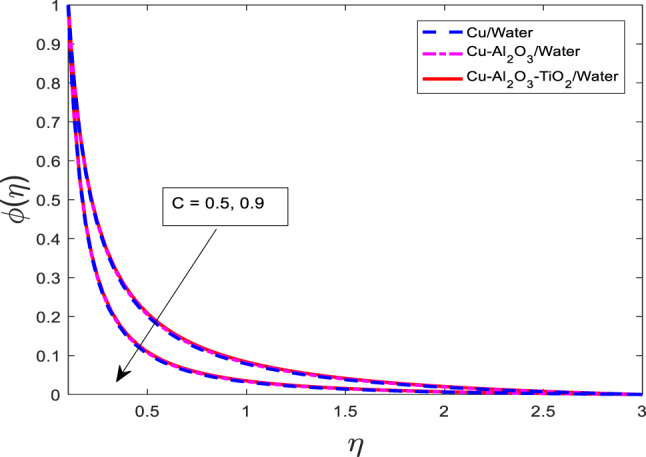
Graphical variations in $$\:\phi\:\:Pr=6.83;\:M=0.5;\:\beta\:=0.1;\:A=0.1;\:B=0.1;\delta\:=1.0;\:$$$$Da=1.1;Sc\:=\:5.1;\:Kr\:=0.1;{\phi}_{1}={\phi}_{2}={\phi}_{3}=0.05$$.

The graphical outcomes of $\:f{^\prime\:}$, $\:\theta\:$, and $\:\phi\:$ for increasing the values of the thickness of the needle $\:c$ are shown in Figs. 18, 19 and 20, respectively. The graphical behavior shows that the velocity field decreases and temperature, along with mass concentration profiles, decrease for all three cases as depicted in Figs. 18, 19 and 20 respectively. Physically, an increase in the thickness of the needle modifies the boundary geometry, which leads to enhanced resistance to the fluid flow and suppresses the velocity field. The thicker needle also alters the thermal and solutal boundary layer structure, resulting in reduced thermal and mass diffusion near the needle surface. Consequently, both the temperature $\:\theta\:$ and concentration $\:\phi\:$ profiles diminish with increasing

### Effect of the thickness of the needle on velocity, temperature, and concentration field

**Fig. 21:**
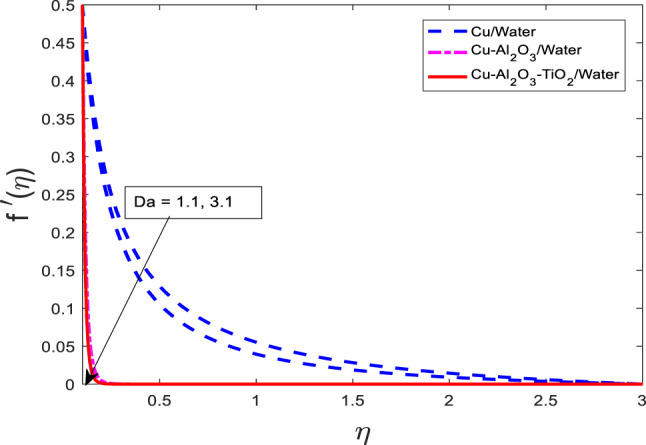
Graphical variations in $$f{^\prime}$$ for $$\:Pr=6.83;\:M=0.5;\beta\:=0.1;\:A=0.1;\:B=0.1;\delta\:=1.0;$$$$c=0.9;\:Sc\:=\:5.1;\:Kr\:=0.1;{\phi}_{1}={\phi}_{2}={\phi}_{3}=0.05$$.

**Fig. 22:**
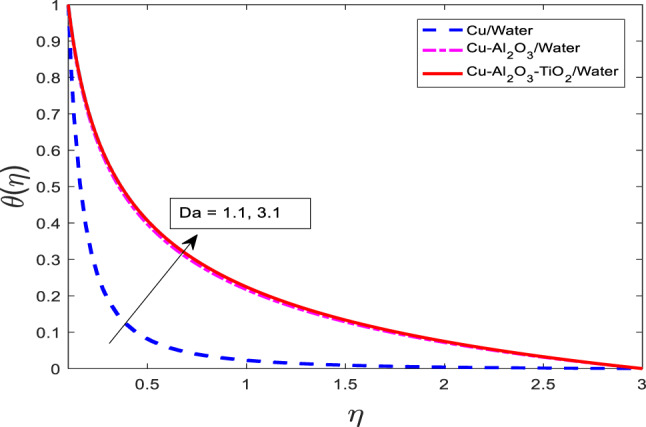
Graphical variations in $$\:\theta\:$$ for $$\:Pr=6.83;\:M=0.5;\beta\:=0.1;\:A=0.1;\:B=0.1;\delta\:=1.0;$$$$c=0.9;\:Sc\:=\:5.1;\:Kr\:=0.1;{\phi}_{1}={\phi}_{2}={\phi}_{3}=0.05$$.

**Fig. 23:**
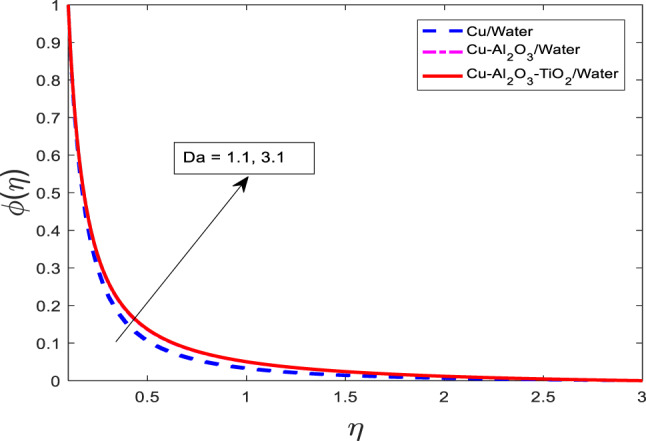
Graphical variations in $$\phi$$ for $$\:Pr=6.83;\:M=0.5;\beta\:=0.1;\:A=0.1;\:B=0.1;\delta\:=1.0;$$$$c=0.9;\:Sc\:=\:5.1;Kr\:=0.1;\:{\phi}_{1}={\phi}_{2}={\phi}_{3}=0.05$$.

The impact of porous media parameters $\:Da$ on for $f{^\prime}$, $\:\theta\:$, and $\:\phi\:$ have been presented in Figs. 21, 22 and 23, respectively. Graphs show that with increasing $\:Da$ for $\:f{^\prime}$ reduces and $\:\theta\:$, and $\:\phi\:$ intensify for all three cases that are nanofluid $\:Cu/$Water, hybrid nanofluid $\:Cu-A{l}_{2}{O}_{3}/$Water, and ternary nanofluid $\:Cu-A{l}_{2}{O}_{3}-Ti{O}_{2}/$Water. Physically, the Darcy number represents the permeability of the porous medium. A higher $\:Da$ implies a more permeable medium, which allows for greater penetration of the fluid through the porous matrix. However, in the context of viscoelastic or nanofluid flow, increased permeability also alters the momentum balance, often introducing additional resistance via drag-like effects, which suppress the fluid velocity as shown in Fig. 21. On the other hand, increased permeability enhances the penetration of thermal and solutal energy into the medium, thereby increasing the temperature and concentration fields. This occurs due to more effective convective transport through the porous structure. Among the fluid types, the ternary nanofluid consistently exhibits the highest $\:\theta\:$ and $\:\phi\:$ values due to its superior thermal and mass transport properties, while the Cu/water nanofluid shows the lowest, following the same trend observed in previous figures.

### Effect of the chemical reaction parameter on velocity, temperature, and concentration field

**Fig. 24:**
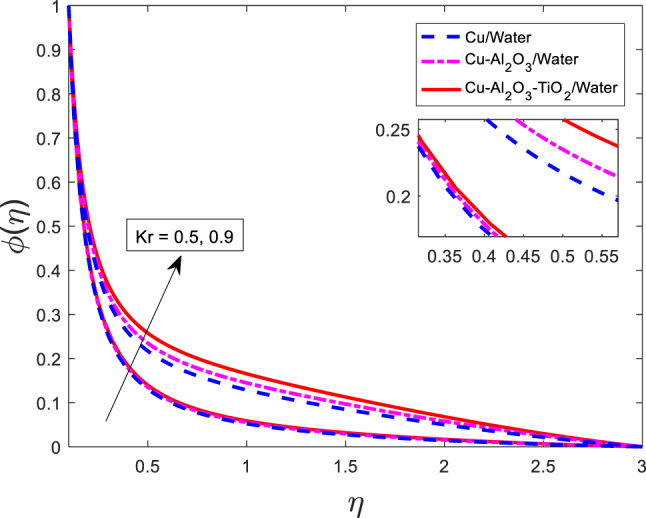
Graphical variations in $$\phi$$ for $$\:Pr=6.83;\:M=0.5;\beta\:=0.1;\:A=0.1;\:B=0.1;$$$$\delta\:=1.0;c=0.9;\:Sc\:=\:5.1;\:{\phi}_{1}={\phi}_{2}={\phi}_{3}=0.05$$.

Figure 24 shows the effects of chemical reaction on mass concentration for all three cases that are nanofluid $\:Cu/$Water, hybrid nanofluid $\:Cu-A{l}_{2}{O}_{3}/$Water, and ternary nanofluid $\:Cu-A{l}_{2}{O}_{3}-Ti{O}_{2}/$Water. The mass concentration increases as $\:Kr$ increases for mono-nanofluid, hybrid nanofluid, and ternary nanofluid. Physically, the parameter Kr​ characterizes the strength of a chemical reaction occurring within the boundary layer. In this context, a positive value of $\:Kr$ corresponds to a generative (constructive) chemical reaction, where species are produced rather than consumed. This leads to an accumulation of solute particles in the fluid, thereby increasing the mass concentration. Among the three nanofluids, the ternary nanofluid exhibits the highest concentration levels due to its enhanced diffusion properties and synergistic behavior from multiple nanoparticle components. In contrast, the Cu/water nanofluid shows the lowest concentration values, as it possesses the least effective transport characteristics. The hybrid nanofluid lies between these two extremes.

### Effect of the chemical reaction parameter on Sherwood number

**Fig. 25:**
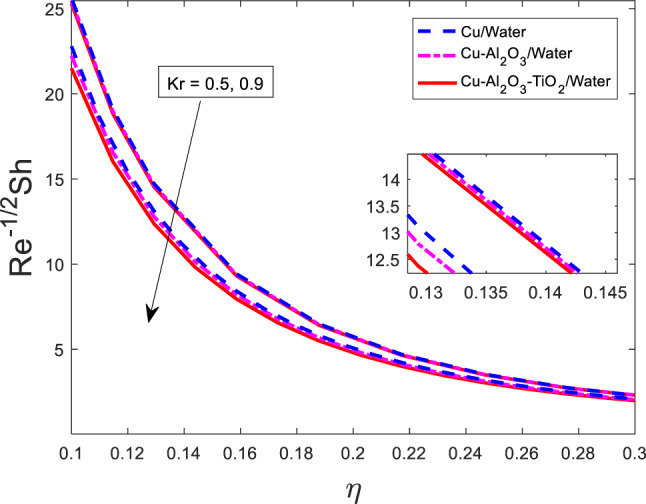
Graphical variations in $$\:R{e}^{-1/2}Sh$$ for $$\:Pr=6.83;\:M=0.5;\beta\:=0.1;\:A=0.1;\:B=0.1;$$$$\delta\:=1.0;\:c=0.9;\:Sc\:=\:5.1;Kr\:=0.1;\:{\phi}_{1}={\phi}_{2}={\phi}_{3}=0.05$$.

Figure 25 shows the impact of chemical reaction on the Sherwood number and it can be seen that the Sherwood number is decreasing as $\:Kr$ is increased for all three types of fluids. Physically, the Sherwood number represents the dimensionless mass transfer rate at the boundary. An increase in the chemical reaction parameter $\:Kr$ assuming it corresponds to a reactive consumption or intensified reaction rate, leads to a reduction in the net mass flux at the surface. This is because the chemical reaction consumes solute species in the boundary layer, diminishing the concentration gradient driving the mass transfer. As a consequence, the Sherwood number decreases. This trend is consistent across the nanofluids, although the exact magnitude of reduction varies due to their differing mass transport properties, with the ternary nanofluid generally maintaining a higher Sherwood number compared to the simpler nanofluid due to its enhanced diffusion characteristics.

### Comparison of the current results with published

**Fig. 26:**
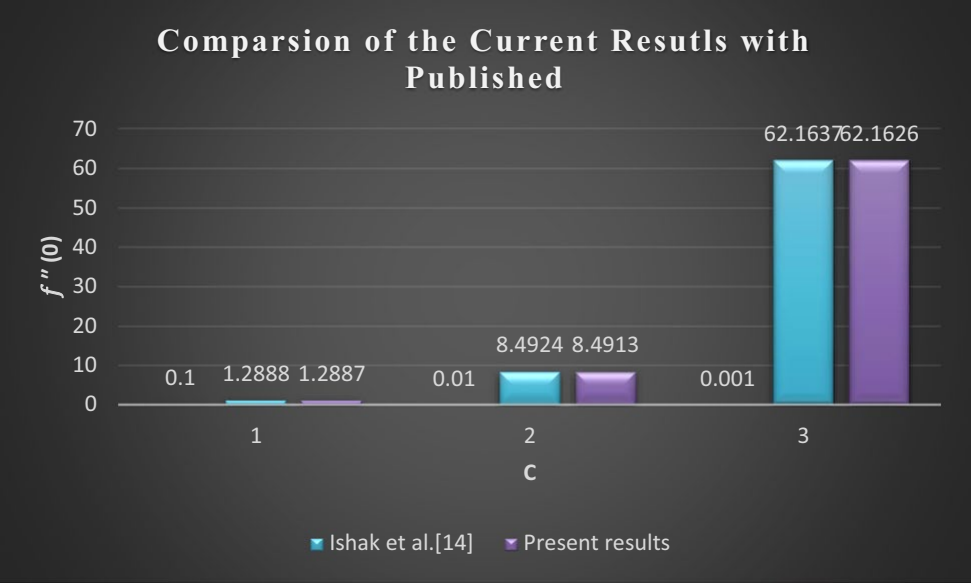
Comparison of the current results with published results.

Figure 26 and Table 2 show the comparison of the currents with those already published. The results show excellent agreement, which reflects the validity of the current results.

**Table 2:** Comparison of $\:{f}^{{\prime}{\prime}}\left(0\right)$, for a specific case.

$\:c$	Ishak et al.^14^	Present
0.1	1.2888	1.2887
0.01	8.4924	8.4913
0.001	62.1637	62.1626

## Conclusion

The study is concentrated on the effects of variable heat rise/fall on magnetohydrodynamic Maxwell ternary nanofluid (copper-alumina-titanium dioxide/water) flow over a moving needle embedded in a porous media under the influence of chemical reaction parameter. The main outcomes of the study from the numerical results are concluded in the following:

- Viscoelastic effects introduce additional resistance, reducing momentum but enhancing thermal and mass diffusion.
- Increased magnetic influence enhances thermal performance, especially in ternary nanofluid. The increasing Lorentz force leads to a rise in temperature and concentration, but the reverse trend in velocity is noted for same parametric conditions.
- Internal heat generation boosts thermal and solutal diffusion.
- Non-uniform heat removal leads to stronger thermal gradients and thicker boundary layers.
- Stronger surface motion intensifies heat and mass transfer.
- Thicker needles introduce more geometric resistance and reduce convective transport.
- Enhanced permeability allows more diffusion but impedes momentum due to drag forces.
- Ternary nanofluid shows the strongest enhancement due to superior diffusive properties.
- Sherwood number decreases as it increases, indicating reduced surface mass transfer.
- Across all cases, the ternary nanofluid Copper-Alumina-Titanium Dioxide/Water) consistently shows superior thermal and mass transfer characteristics.
- The mono-nanofluid Cu/water shows the least enhancement, with the hybrid nanofluid lying in between.
- As the volume fractions of nanoparticles increase, the effective viscosity and density of the nanofluids also rise, leading to a noticeable decline in velocity. Conversely, the temperature and concentration fields intensify due to the improved thermal conductivity and augmented mass diffusion capabilities of the nanofluids.
- A comparison between the current findings and previously published results demonstrates strong agreement, confirming the validity and accuracy of the present computations.

**Future Recommendations/Directions**.

- The current probleµ can be extended to investigate unsteady (transient) versions of the problem to model more realistic industrial or biomedical processes where flow and thermal conditions change with time.
- The current model can be extended to explore the influence of spatially or temporally varying magnetic fields to capture effects more relevant to magnetic drug targeting or electromagnetic processing.
- It can be studied to incorporate radiative heat transfer effects to enhance the model’s applicability to high-temperature environments such as solar collectors or nuclear reactors.
- It can be extended to the model to 3D geometries, such as rotating or conical needles, to match complex engineering designs and improve prediction capabilities.
- It can conduct an experimental study or use machine learning-based surrogate models to validate the theoretical predictions and speed up parameter optimization.
- The current model can be extended to explore bio-convective flow or drug delivery scenarios by incorporating motile microorganisms or biocompatible ternary nanofluids within the current framework.

## Data Availability

All the data generated or analysed during this study are included in the article.
